# Early rhythm control compared to rate control in atrial fibrillation – A systematic review, meta-analysis, and meta-regression

**DOI:** 10.1016/j.ipej.2025.02.003

**Published:** 2025-02-12

**Authors:** Katherine Hermanto, Raymond Pranata, Hawani Sasmaya Prameswari, Giky Karwiky, Chaerul Achmad, Mohammad Iqbal

**Affiliations:** Department of Cardiology and Vascular Medicine, Faculty of Medicine, Universitas Padjadjaran, Hasan Sadikin General Hospital, Bandung, Indonesia

**Keywords:** Atrial fibrillation, Early rhythm control, Rate control, Ablation, Meta-analysis

## Abstract

**Background:**

This meta-analysis aimed to compare the effectiveness of early rhythm control to rate control, and whether catheter ablation derived more benefit compared to other methods of rhythm control.

**Methods:**

A comprehensive literature search was conducted on PubMed, SCOPUS, and EuropePMC up to July 2, 2024. The primary outcome of this study was major adverse cardio-cerebrovascular events (MACCE), defined as a composite of mortality, stroke/systemic embolism, heart failure hospitalization (HFH), and acute coronary syndrome (ACS) during the follow-up period. Outcome measures were adjusted hazard ratios (aHR).

**Results:**

A total of 504,124 patients from 11 studies were included in this systematic review and meta-analysis. Early rhythm control was significantly associated with reduction in MACCE (aHR 0.85 [95 % CI 0.80, 0.90], p < 0.001; I^2^: 23 %), stroke (aHR 0.79 [95 % CI 0.72, 0.86], p < 0.001; I^2^: 25 %), HFH (aHR 0.87 [95 % CI 0.78, 0.96], p = 0.008; I^2^: 48 %), and ACS (aHR 0.80 [95 % CI 0.66, 0.96], p = 0.018; I^2^: 40 %). No mortality benefit (aHR 0.93 [95 % CI 0.85, 1.01], p = 0.066; I^2^: 67 %) was observed; however, mortality benefit became evident (aHR 0.87 [95 % CI 0.85, 0.89], p < 0.001) upon removal of a study during a leave-one-out sensitivity analysis. Meta-regression analysis showed that the benefits of early rhythm control in terms of MACCE were more pronounced with ablation (coefficient −0.004, p = 0.010, R^2^: 100 %).

**Conclusion:**

Early rhythm control was associated with better outcomes compared to rate control in AF, with a more pronounced benefit observed for ablation.

## 1 Introduction

Atrial fibrillation (AF) is the most common sustained cardiac arrhythmia in the general population and is associated with increased mortality and morbidity [1]. Previous randomized trials comparing rhythm-control and rate-control strategies, including the landmark AFFIRM (Atrial Fibrillation Follow-up Investigation of Sinus Rhythm Management), reported no significant differences between these treatment strategies in terms of mortality and stroke incidence. However, recent studies have shown that rhythm control is associated with a lower risk of adverse cardio-cerebrovascular outcomes than rate control in patients with recently diagnosed AF (within 1 year) [1,2]. The EAST-AFNET 4 (Early Treatment of Atrial Fibrillation for Stroke Prevention Trial) revealed that patients randomly assigned to early rhythm control had a lower risk of cardiovascular death, stroke, and hospitalization for worsening heart failure or acute coronary syndrome [2,3].

Catheter ablation (CA) is a viable and safe therapeutic option that potentially outperforms antiarrhythmic drugs (AAD) [4,5]. Moreover, ablation seems to reduce the progression from paroxysmal AF to persistent AF and improves quality of life compared to AAD [4]. Despite this, the use of CA as a rhythm control remained low because it was specifically intended for certain favorable populations [6,7]. Through a systematic review and meta-analysis of existing studies, this study aimed to compare the effectiveness of early rhythm control to rate control and determine whether catheter ablation provides more benefit compared to other methods of rhythm control.

## 2 Methods

### 2.1 Literature search strategy

Two reviewers independently conducted a comprehensive literature search using the specified keywords (((Early rhythm control) or (early ablation) or (early anti arrhythmia) or (early antiarrhythmics)) and (rate control) and (atrial fibrillation))) in PubMed, SCOPUS, and EuropePMC up to 02 July 2024. Discrepancies that emerged were resolved through discussions. Records were assessed for eligibility using predetermined inclusion and exclusion criteria. This systematic review was performed according to the Preferred Reporting Items for Systematic Reviews and Meta-Analyses (PRISMA) guidelines. The protocol for this study is registered in the PROSPERO (CRD42024564592).

### 2.2 Intervention and control groups

The intervention group included AF patients undergoing early rhythm control, defined as receiving rhythm control within 12 months of AF diagnosis as initial treatment. The control group comprised patients receiving rate control as initial treatment.

### 2.3 Selection criteria

We included randomized controlled trials and observational studies (both prospective and retrospective) comparing early rhythm control to rate control as initial treatment in patients with AF. Excluded were animal studies, letters to editors, supplementary journals, review articles, case reports, and non-English language articles.

### 2.4 Outcomes

The primary outcome of this study is major adverse cardio-cerebrovascular event (MACCE), defined as a composite of mortality, stroke/systemic embolism, heart failure hospitalization (HFH), and acute coronary syndrome (ACS) during the follow-up period. Secondary outcomes included mortality, stroke/systemic embolism, HFH, and ACS during the follow-up period. Outcome measures were adjusted hazard ratios (aHR).

### 2.5 Data extraction

Two reviewers independently extracted data using a form detailing primary and secondary outcomes, study design, sample size, inclusion criteria, follow-up duration, age, male sex, left atrial size (volume/diameter), left ventricular ejection fraction (LVEF), hypertension, diabetes, congestive heart failure, coronary artery disease, myocardial infarction, and the percentage of patients receiving ablation, anti-arrhythmic drugs, and cardioversion. The risk of bias was assessed using the Newcastle-Ottawa Scale for observational studies and the Cochrane Risk of Bias tool for randomized controlled trials. Any disagreements were resolved through discussion.

### 2.6 Statistical analysis

This meta-analysis was performed using STATA 17 with a random-effects model, regardless of heterogeneity, using the DerSimonian–Laird method. Statistically significant heterogeneity was defined as an I^2^ value > 50 % and/or a p-value for heterogeneity below 0.10. The effect estimate was considered statistically significant when the p-value was less than 0.05. Adjusted hazard ratios were pooled from the included studies. Egger’s test was used to quantitatively measure small-study effects, and funnel-plot analysis followed by trim-and-fill analysis was used to qualitatively measure small-study effects and publication bias. A meta-regression analysis using the DerSimonian–Laird approach examined the effect of moderating variables on the primary outcome, focusing on baseline factors such as age, male sex, BMI, paroxysmal AF, HF, diabetes, hypertension, stroke, and ablation. Only variables reported by at least five studies were analyzed. Leave-one-out sensitivity analyses were performed to assess the robustness of the analyses and explore causes of heterogeneity.

## 3 Results

**Fig. 1:**
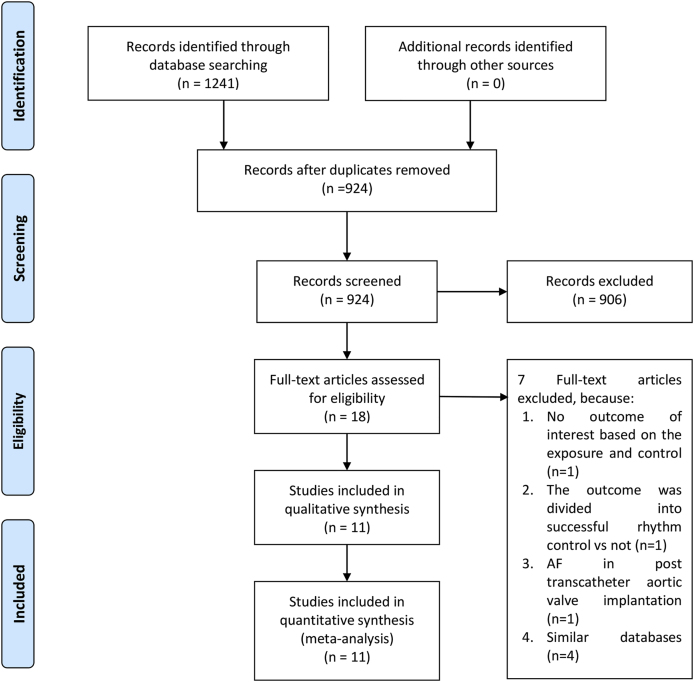
PRISMA flowchart.

Two RCTs, one retrospective analysis of RCT, and eight observational studies were identified. A total of 504,124 patients were included in this systematic review and meta-analysis [Fig. 1] [[8], [9], [10], [11], [12], [13], [14], [15], [16], [17], [18]]. All of the studies included in this systematic review and meta-analysis provided data regarding AAD as rhythm control strategy and eight studies provided data regarding CA as rhythm control strategy. The baseline characteristics of the studies are presented in Table 1, Table 2.

**Table 1:** Baseline characteristics of the included studies.

Studies (Author)	Age (years)	Male (%)	BMI (kg/m^2^)	Paroxysmal (%)	HF (%)	DM (%)	HTN (%)	CAD (%)	MI (%)	Stroke (%)	LA d (mm)	LAVI (mm/m^2^)	LVEF (%)
Blomström-Lundqvist et al., 2020	71.86	53.3	>30 kg/m^2^: 30.77	N/A	26.3	N/A	85.3	29.6	N/A	N/A	LA d > 40 mm: 66.56	N/A	LVEF <35 %: 4.19
Chao et al., 2022	69.44	56.3	N/A	N/A	24.4	26.6	64.7	N/A	N/A	20.7	N/A	N/A	N/A
Dickow et al., 2022	70.1	59.7	N/A	N/A	25.7	44.3	92.2	74.9	34	20.1	N/A	N/A	N/A
Girod et al., 2022	66	74.2	27	61.5	20.6	6.5	57.5	20	N/A	14.6	N/A	N/A	N/A
Kany et al., 2022	69	57.5	28.56	N/A	52.5	17.3	91.8	38.7	N/A	8.79	N/A	N/A	N/A
Kim et al., 2021	70	54.7	N/A	N/A	54.9	29.6	80.3	N/A	8,6	35.8	N/A	N/A	N/A
Kirchhof et al., 2020	70.3	53.7	29.3	35.7	28.6	N/A	87.8	N/A	N/A	11.76	N/A	N/A	N/A
Park et al., 2022	69.31	61.9	24.3	52.0	7.3	32.2	67.8	N/A	N/A	N/A	N/A	43.3	62.7
Pope et al., 2023	Median 73 & 68	55.7	Median 26.5 & 27.1	31.5	21.8	22.1	75.9	10.7	N/A	10.9	N/A	N/A	N/A
Proietti et al., 2021	69.77	53.7	27.71	40.8	37.5	23.4	73.2	10.6	N/A	10.5	N/A	N/A	N/A
Yang et al., 2020	71	39.2	28.72	N/A	N/A	20.4	71.2	41.7	17.6	N/A	4.33	N/A	55.8

BMI: body mass index, HF: heart failure, DM: diabetes mellitus, HTN: hypertension, CAD: coronary artery disease, MI: myocardial infarction, LA d: left atrial diameter, LAVI: left atrial volume index, LVEF: left ventricle ejection fraction.

**Table 2:** Baseline characteristics of the included studies (continued).

Studies (Author)	Study design	Sample size (n)	Patient’s characteristic	Paroxysmal	Rhythm Control	Follow up (years)	Ablation (%)	AAD (%)	Cardioversion (%)	Rate control (%)	NOS
Blomström-Lundqvist et al., 2020	Post Hoc Analysis of randomized controlled trial (ATHENA)	2859	Patients with a documented history of AF/AFL Patients aged 75 years or older with at least one risk factor for cardiovascular events. Patients aged 70 years or older with no more than one risk factor for cardiovascular events. Patients aged 65 years or older with at least two risk factors for cardiovascular events.	N/A	Dronedarone	1	2.97	62.6	N/A	N/A	8
Chao et al., 2022	Propensity-Score Matched Prospective Cohort Study	301,064	AF patients aged 20 years from January 1, 2001 to December 31, 2016	N/A	AADs or catheter ablation	Median: 5.1	0.9	20.73	0.54	45.27	8
Dickow et al., 2022	Retrospective Cohort Study	109,739	All adult patients diagnosed with AF aged >75 years or had a previous TIA or stroke, or met 2 of the following criteria: age >65 years, female sex, HF, HTN, DM, severe CAD, CKD	N/A	AAD: 96.3 Catheter ablation: 8.4	2.6	9.1	47.5	32.9	N/A	8
Girod et al., 2022	Prospective Cohort Study	2518	Documented AF	61.6	PVI and non-PVI	Median 3.9	100	N/A	N/A	N/A	8
Kany et al., 2022	Propensity-Score Matched Prospective Cohort Study (UK Biobank)	9004	Diagnosis of AF within 1 year and presence of cardiovascular conditions and risk factors, namely age >75 years, a previous stroke or TIA, or two of the following: age >65 years, female sex, HTN, DM, severe CAD, HF, CKD or PAD.	N/A	AAD therapy (amiodarone, dronedarone, flecainide, propafenone or sotalol) or AF ablation	Early rhythm control group: 4.94 Usual care group: 2.54	11	N/A	N/A	N/A	8
Kim et al., 2021	Prospective Cohort Study	22,635	Patients with AF and treated for rhythm control older than 75 and had history of TIA or stroke OR met 2 of: age >65, female sex, HF, HTN, DM, previous MI, or CKD	N/A	Early rhythm control: AF ablation: 1.6 Amiodarone: 39.9 Dronedarone: 2.0 Flecanide: 24.9 Pilsicanide: 4.0 Propafenone: 25.5 Sotalol: 2.1 Late rhythm control: AF ablation: 14.5 Amiodarone: 41.4 Dronedarone: 1.7 Flecanide: 18.7 Pilsicanide: 4.7 Propafenone: 17.1 Sotalol: 1.9	2.1	1.6	N/A	N/A	N/A	8
Kirchhof et al., 2020	Open label RCT (EAST-AFNET 4)	2789	Adults with early AF, ≥75 years of age, female sex, HF, HTN, DM, severe CAD, CKD	Early rhythm control: 36.0 usual care group: 35.4	In the early rhythm control group, almost all patients (94.8 %) received either an antiarrhythmic drug or underwent atrial fibrillation ablation. After 2 years, 65.1 % of patients were still receiving active rhythm-control therapy. Specifically, 270 patients were treated with atrial fibrillation ablation, and 638 patients were treated with antiarrhythmic drugs	Median: 5.1	8	86.8	39	N/A	Low risk of bias apart from no masking^a^
Park et al., 2022 (RAFAS Trial)	Open Label RCT	300	Patients aged 20–80 years who were hospitalized for an acute stroke, patients for whom AF was diagnosed through intensive ECG monitoring for at least 72 h were enrolled with the consent of the patient or guardian	51.2	AAD	1	12.4	N/A	15.5	N/A	Low risk of bias apart from no masking^a^
Pope et al., 2023	Prospective Observational Study	45,382	Patients were diagnosed with AF within six weeks - on average two weeks - before enrolment, and had at least one risk factor for stroke.	rhythm control group: 25.6 rate control group: 36.9	AAD, cardioversion, or ablation – alone or in combination with rate modifiers	2	0.7	N/A	N/A	N/A	9
Proietti et al., 2021	Prospective Observational Study (EHRA-ESC EORP-AF General Long-Term Registry)	3774	AF documented within 12 months before enrollment on the basis of objective ECG evaluation.	38.5	Early rhythm control: Drugs: 28.2 ECV: 22.5 PCV: 9.9 CA: 3.3 mixed strategies: 36.1	1 and 2	3.3; mixed 36.1	9.9	22,5	N/A	9
Yang et al., 2020	Retrospective Cohort Study (AFFIRM substudy)	4060	Clinical documentation of at least 6 h of AF within 6 months preceding enrollment	N/A	Amiodarone, sotalol, or class I antiarrhythmic drug	>5	0.9 (initially 0)	99.1	N/A	N/A	8

AAD: antiarrhythmic drug, NOS: Newcastle Ottawa Scale, RCT: randomized controlled trial, EAST-AFNET 4: The Early Treatment of Atrial Fibrillation for Stroke Prevention Trial, AF: atrial fibrillation, HF: heart failure, HTN: hypertension, DM: diabetes mellitus, CAD: coronary artery disease, CKD: chronic kidney disease, ATHENA: , AFL: atrial flutter, AFFIRM: Atrial Fibrillation Follow-up Investigation of Rhythm Management, ECG: electrocardiography, ECV: electric cardioversion, PCV: pharmacological cardioversion, CA: catheter ablation, TIA: transient ischemic attack, RAFAS: , PVI: pulmonary vein isolation.

a Based on Cochrane risk of bias.

### 3.1 Outcomes

**Fig. 2:**
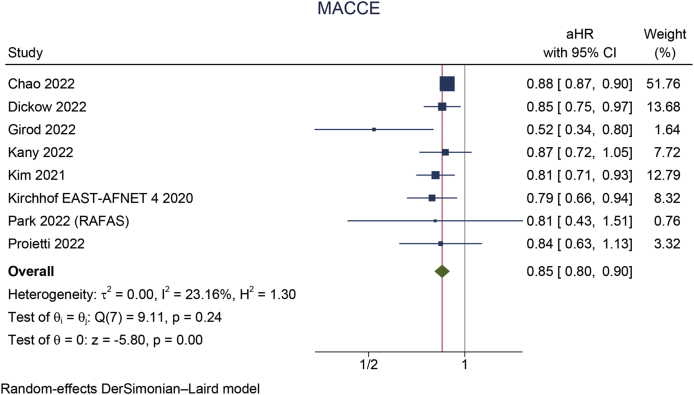

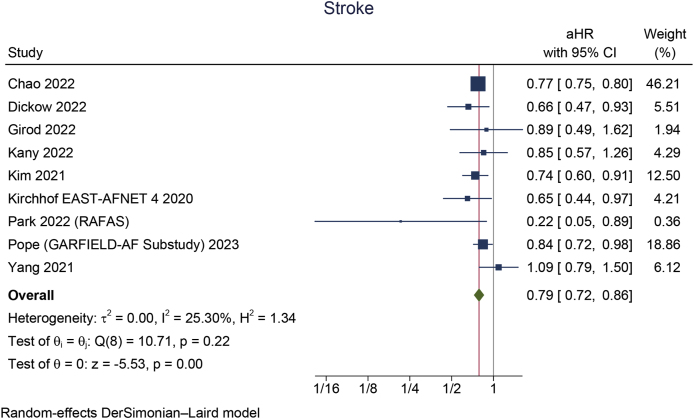

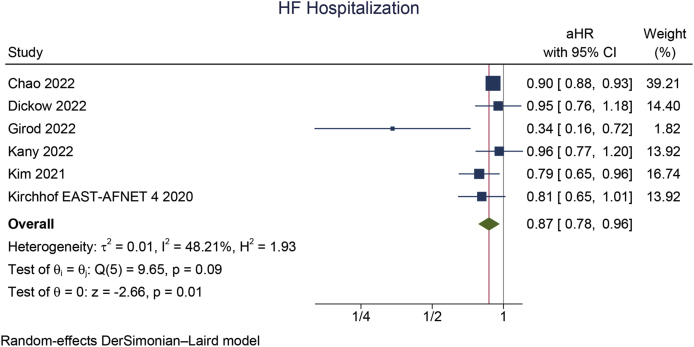
Early rhythm control versus rate control in terms of A) MACCE, B) stroke, and C) heart failure hospitalization. MACCE: major adverse cardio-cerebrovascular events.

**Fig. 3:**
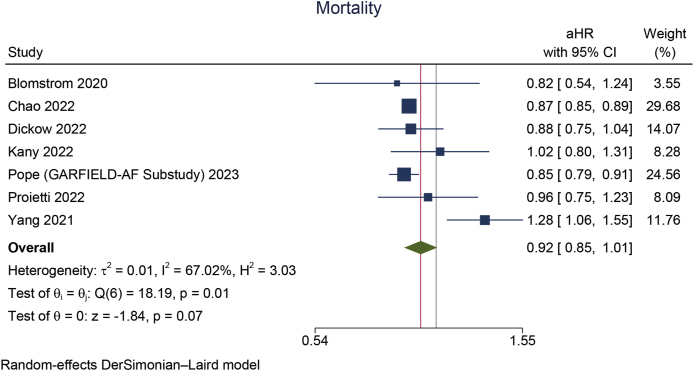

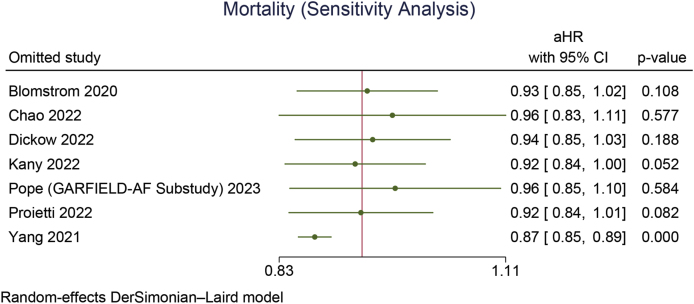
Early rhythm control versus rate control in terms of A) mortality and B) leave-one-out sensitivity analysis for mortality.

Early rhythm control was significantly associated with a reduction in MACCE (aHR 0.85 [95 % CI 0.80, 0.90], p < 0.001; I^2^: 23 %, p = 0.245 [Fig. 2A]), stroke (aHR 0.79 [95 % CI 0.72, 0.86], p < 0.001; I^2^: 25 %, p = 0.219 [Fig. 2B]), HFH (aHR 0.87 [95 % CI 0.78, 0.96], p = 0.008; I^2^: 48 %, p = 0.086 [Fig. 2C]), and ACS (aHR 0.80 [95 % CI 0.66, 0.96], p = 0.018; I^2^: 40 %, p = 0.138). However, early rhythm control was not significantly associated with a change in mortality (aHR 0.93 [95 % CI 0.85, 1.01], p = 0.066; I^2^: 67 %, p = 0.006 [Fig. 3A]).

### 3.2 Subgroup analysis by study design

Subgroup analysis for RCT showed that early rhythm control was significantly associated with a reduction in MACCE (aHR 0.79 [95 % CI 0.67, 0.94], p < 0.001; I^2^: 0 %, p = 0.945), stroke (aHR 0.47 [95 % CI 0.17, 1.26], p = 0.329; I^2^: 53.8 %, p = 0.329). Subgroup analysis for observational studies showed that early rhythm control was significantly associated with a reduction in MACCE (aHR 0.85 [95 % CI 0.80, 0.91], p = 0.002; I^2^: 34.8 %, p = 0.175), stroke (aHR 0.79 [95 % CI 0.74, 0.84], p = 0.001; I^2^: 11.4 %, p = 0.343).

### 3.3 Leave-one-out sensitivity analysis

Leave-one-out sensitivity analyses indicated that early rhythm control remained significantly associated with reductions in MACCE, stroke, HF hospitalization, and ACS (all p < 0.05). Mortality reduction became significant with early rhythm control (aHR 0.87 [95 % CI 0.85, 0.89], p < 0.001) upon the removal of the Yang 2021 study [Fig. 3B].

### 3.4 Meta regression MACCE (primary outcome)

**Fig. 4:**
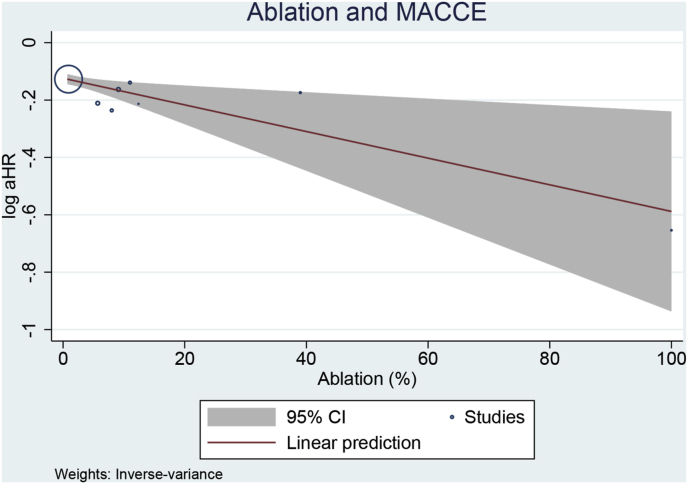
Bubble plot showing the benefits of early rhythm control in terms of MACCE were more pronounced in patients receiving ablation.

Meta-regression analysis showed that the benefits of early rhythm control in terms of MACCE were more pronounced in patients receiving ablation (coefficient −0.004, p = 0.010, R^2^: 100 % [Fig. 4]), with ablation patients receiving a larger magnitude of benefit compared to other modalities. Age (p = 0.325), male (p = 0.087), BMI (p = 0.420), paroxysmal AF (p = 0.111), HF (p = 0.794), diabetes (p = 0.495), hypertension (p = 0.892), and stroke (p = 0.998) did not significantly influence the outcome.

### 3.5 Publication bias

**Fig. 5:**
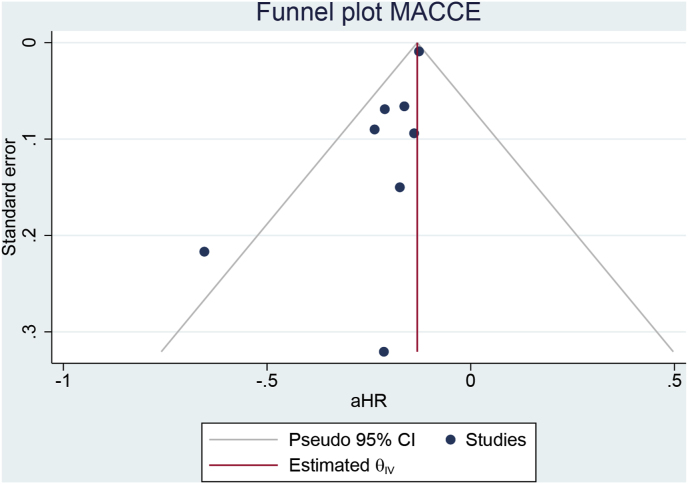

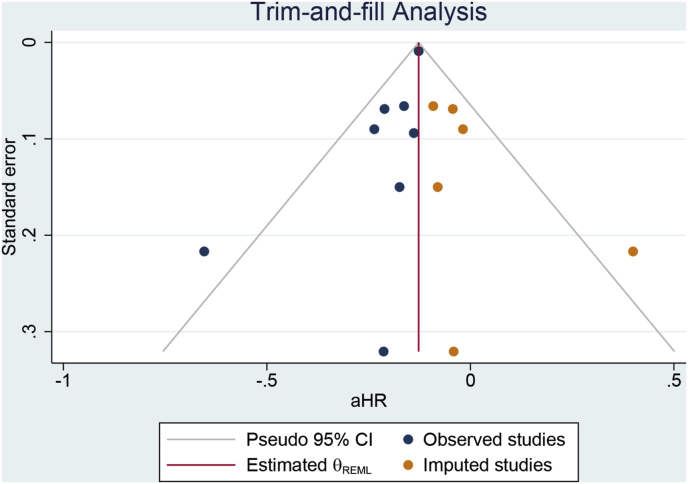
Publication bias analysis for MACCE. A) funnel plot analysis and B) trim-and-fill analysis.

Egger’s test was significant for small-study effects for the MACCE outcome (p = 0.023); the funnel plot was asymmetrical [Fig. 5A]. Non-parametric trim-and-fill analysis showed that by imputing 9 entries [Run R0, imputation on the right side], the benefit of early rhythm control remained consistent (aHR 0.88 [95 % CI 0.87, 0.90] [Fig. 5B]). Egger’s test was not significant for small-study effects for the stroke outcome (p = 0.547), with a relatively symmetrical funnel plot except for one outlier on the left side. Egger’s test was not significant for small-study effects for the mortality outcome (p = 0.439), with a relatively symmetrical funnel plot except for one outlier on the right side. Egger’s test was not significant for small-study effects for the HF hospitalization outcome (p = 0.076), with a relatively symmetrical funnel plot except for one outlier on the left side. Egger’s test was significant for small-study effects for the ACS outcome (p = 0.006); the funnel plot was asymmetrical, and non-parametric trim-and-fill analysis showed that by imputing 9 entries [Run R0, imputation on the right side], the benefit of early rhythm control remained consistent (aHR 0.92 [95 % CI 0.89, 0.95]). The risk of bias in the observational studies was low, with each study scoring ≥7 based on NOS [Table 2]. Similarly, the risk of bias in the RCTs was also low, except for the lack of masking due to their open-label design [Supplementary Fig. 1].

## 4 Discussion

The main finding of our analysis was that MACCE significantly decreased in patients with AF receiving early rhythm control. However, early rhythm control was not significantly associated with a change in mortality. These results were consistent across most of the studies analyzed. An important additional finding was that catheter ablation as an early rhythm control method showed a significant reduction in MACCE.

Unexpected results of the AFFIRM study led professional society guidelines to recommend rate control as the first-line therapy for recurrent forms of AF (paroxysmal and persistent AF). However, following the results of the EAST-AFNET 4 trial, physicians conducted several recent studies that countered the earlier findings [19]. Most of the analyzed studies reported a lower incidence of stroke, heart failure hospitalization (HFH), and acute coronary syndrome (ACS) [4,20]. Regarding the hemodynamic and physiological consequences, normal sinus rhythm was found to be better than AF [20]. AF is usually considered a progressive disorder, beginning as paroxysmal AF and advancing to persistent and then “permanent” AF with electrical and structural remodeling of the atrium [20,21]. Structural, electrical, and autonomic remodeling are all affected by the arrhythmia, which can exacerbate pre-existing issues and make patients more susceptible to recurring and chronic AF [20]. Therefore, maintaining sinus rhythm as early as possible in the natural history of AF appears to be a rational approach to preventing AF progression [3].

Our study supports the notion that rhythm control within one year of AF diagnosis can significantly reducing MACCE (stroke, HFH, and ACS). Although the pooled analysis showed no mortality benefit, the advantage became evident upon removal of a study in a leave-one-out sensitivity analysis. The excluded study which also an AFFIRM substudy, demonstrated that more than half of the AFFIRM study participants met the EAST-AFNET 4 trial’s definition of early AF. Stratified analysis of AFFIRM participants with AF diagnosed within 6 months before study enrollment did not reveal any significant clinical benefit of rhythm control over rate control in an intention-to-treat analysis. These findings suggest no significant difference in clinical response to AF treatment strategies between new and old AF groups. This may be explained by the use of different treatment options between AFFIRM and EAST-AFNET 4 such as increased use of B-blockers, flecainide, and dronedarone and decreased use of digoxin and sotalol. In AFFIRM, warfarin was discontinued in patients assigned to rhythm control. However, the patients in both arms of the EAST-AFNET 4 trial continued to use oral anticoagulation. And last, unlike EAST-AFNET 4 study, CA was not used in the AFFIRM study population. It indicated that CA plays important role in improvement of the outcomes of patients assigned to rhythm control in EAST-AFNET 4 study [1].

The optimal rhythm control therapy strategy is still a matter of debate. A recently published retrospective cohort study successfully proved the association between early ablation and lower risk of AF recurrency in patients with paroxysmal AF, persistent AF and in elderly patients [4]. Shorter diagnosis-to-ablation time will have a less severe atrial remodeling compared to longer diagnosis-to-ablation time and has a lower chance of AF recurrence after conversion to sinus rhythm [20,22]. In certain populations, CA showed significant benefit not only in mortality benefit but also improvement in quality of life. Patients <65 years of age derive more benefit from CA due to high burden of comorbidities and procedural complications in older patients. In patients with heart failure reduced ejection fraction (HFrEF), early rhythm control using CA showed improvement in cardiac hemodynamics because patients with HFrEF are more dependent on atrial contraction for maintaining adequate cardiac output as well as reduction in the incidence of tachycardia-mediated cardiomyopathy [23]. In contrast, few studies failed to show a significant benefit of CA over medical therapy mostly in elderly population, persistent/longstanding persistent AF with HFrEF, and patients with more advanced left atrial remodeling [24]. The studies analyzed in this meta-analysis has limited data regarding EF, left atrial diameter, and left atrial volume index. Indeed, meta-regression analysis indicated that patients receiving catheter ablation derived more benefits compared to those receiving early rhythm control using other modalities, thus expanding the role of catheter ablation in early presenters.

### 4.1 Clinical implications

This meta-analysis supports prioritizing early rhythm control in treating AF patients. Early rhythm control leads to a reduction in MACCE, stroke, HFH, and ACS. Additionally, catheter ablation provided more benefits compared to antiarrhythmic drugs or cardioversion, suggesting that early rhythm control using catheter ablation can be considered for patients with favorable clinical profiles. In patients with suspected tachycardia-induced cardiomyopathy, catheter ablation can be considered the first choice of treatment.

### 4.2 Limitations

Our study has certain limitations. The definition of MACCE outcomes varied across the included studies. While associations were analyzed, causality was not assessed. Although most data were obtained from observational studies, these studies generally had a low risk of bias and a large sample size. Due to limited number of studies, the subgroup analyses for RCTs were only based on two studies. Future randomized controlled trials are necessary to directly compare the outcomes of early rhythm control through ablation with those achieved using other approaches. Limitations inherent to the random-effects model and the observed heterogeneity also need to be acknowledged. Furthermore, variables such as left atrial size, left ventricular hypertrophy, and LVEF were not consistently reported across studies, preventing meta-regression analysis that could identify patient subgroups more likely to benefit from early rhythm control.

## 5 Conclusion

Early rhythm control is better in reducing MACCE compared to rate control in patients with AF. Early rhythm control by ablation showed lower incidence of MACCE in patients in majority of the studies compared to patients receiving AAD or cardioversion as early rhythm control.

## Patient consent statement

N/A.

## Authors' contributions

KH: Conceptualization, Data Curation, Investigation, Formal Analysis, Writing – Original Draft, Writing – Review and Editing.

RP: Conceptualization, Data Curation, Investigation, Formal Analysis, Methodology, Supervision, Writing – Original Draft.

HSP: Data Curation, Investigation, Supervision, Writing – Review and Editing.

CA: Data Curation, Investigation, Writing – Review and Editing.

GK: Data Curation, Investigation, Writing – Review and Editing.

MI: Conceptualization, Methodology, Supervision, Writing – Original Draft, Writing – Review and Editing.

## Data availability

Available on reasonable request.

## Ethical approval

N/A.

## PROSPERO registration

CRD42024564592.

## Funding

None.

## Declaration of competing interest

The authors declare that they have no known competing financial interests or personal relationships that could have appeared to influence the work reported in this paper.
