# Supplementary material for: Early rhythm control compared to rate control in atrial fibrillation – A systematic review, meta-analysis, and meta-regression
Source: Indian Pacing Electrophysiol J. 2025 Feb 12;25(2):82–90. doi: 10.1016/j.ipej.2025.02.003 (PMC12138061; doi:10.1016/j.ipej.2025.02.003)
Supplement: Multimedia component 1 [file mmc1.docx]

Supplementary Table 1.

| **Database** | **Keywords** | **Results** |
| --- | --- | --- |
| PubMed | (((Early rhythm control) or (early ablation) or (early anti arrhythmia) or (early antiarrhythmics)) and (rate control) and (atrial fibrillation))) | 443 |
| SCOPUS | ( ( early AND rhythm AND control ) OR ( early AND ablation ) OR ( early AND anti AND arrhythmia ) OR ( early AND antiarrhythmics ) ) AND ( rate AND control ) AND ( atrial AND fibrillation )) | 597 |
| EuropePMC | (((Early rhythm control) or (early ablation) or (early anti arrhythmia) or (early antiarrhythmics)) and (rate control) and (atrial fibrillation))) | 221 |

| **Selection** | Blomström-Lundqvist et al 2020 | Chao et al 2022 | Dickow et al 2022 | Girod et al 2022 | Kany et al 2022 | Kim et al 2021 | Pope et al 2023 | Proietti et al 2021 | Yang et al 2020 |
| --- | --- | --- | --- | --- | --- | --- | --- | --- | --- |
| Representative of the cohort? | * | * | * | * | * | * | * | * | * |
| Selection of the non-exposed | * | * | * | * | * | * | * | * | * |
| Ascertainment of exposure | * | * | * | * | * | * | * | * | * |
| Demonstration that outcome  was not present at start of study | * | * | * | * | * | * | * | * | * |
| **Comparability** |  |  |  |  |  |  |  |  |  |
| Comparability of cohorts on the basis of the design or analysis | * | * | * | * | * | * | ** | ** | * |
| **Outcome** |  |  |  |  |  |  |  |  |  |
| Assessment of outcome | * | * | * | * | * | * | * | * | * |
| Was follow up long enough for outcomes to occur | * | * | * | * | * | * | * | * | * |
| Adequacy of follow up of cohorts | * | * | * | * | * | * | * | * | * |
| Total quality score | 8 | 8 | 8 | 8 | 8 | 8 | 9 | 9 | 8 |

Supplementary Table 2. Newcastle-Ottawa Scale

Supplementary Table 2. Cochrane Risk of Bias Assessment Tool


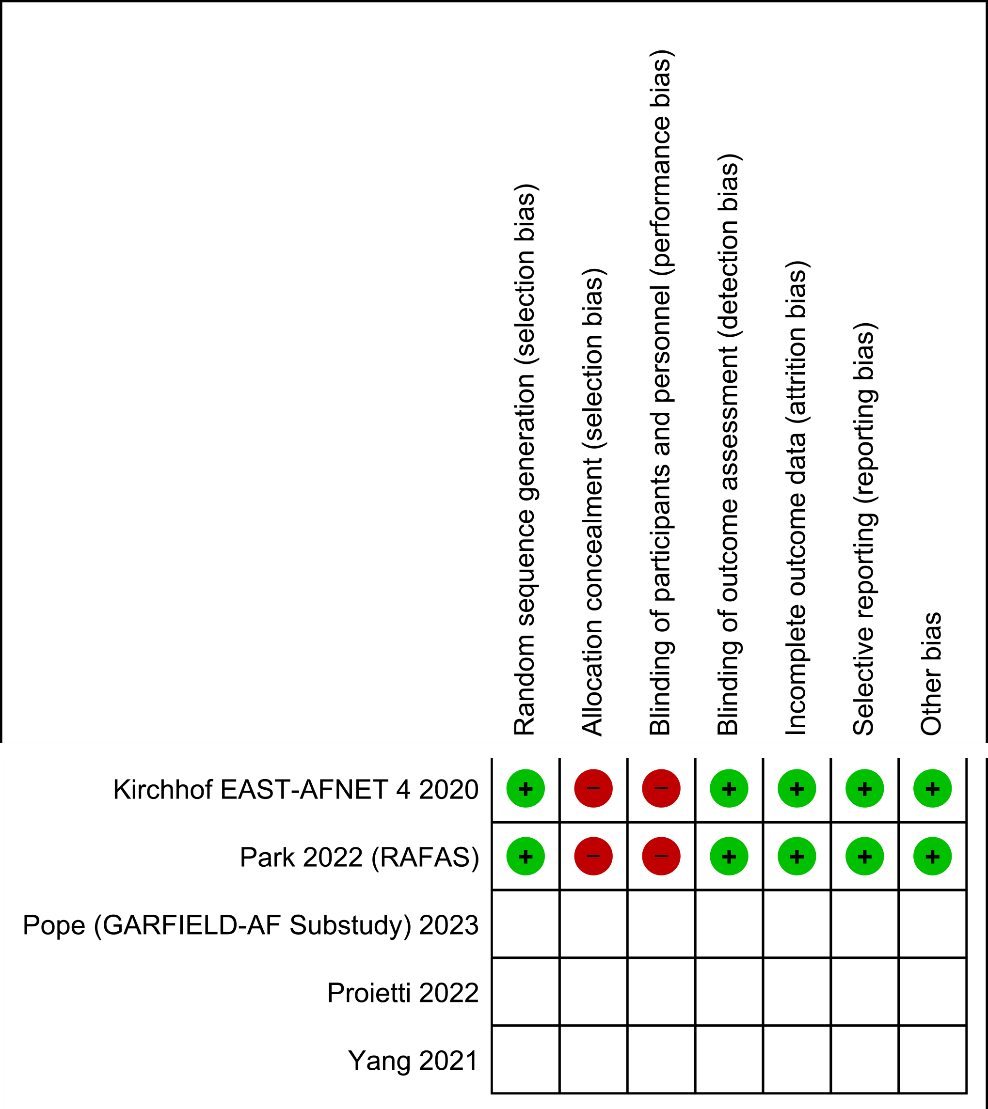


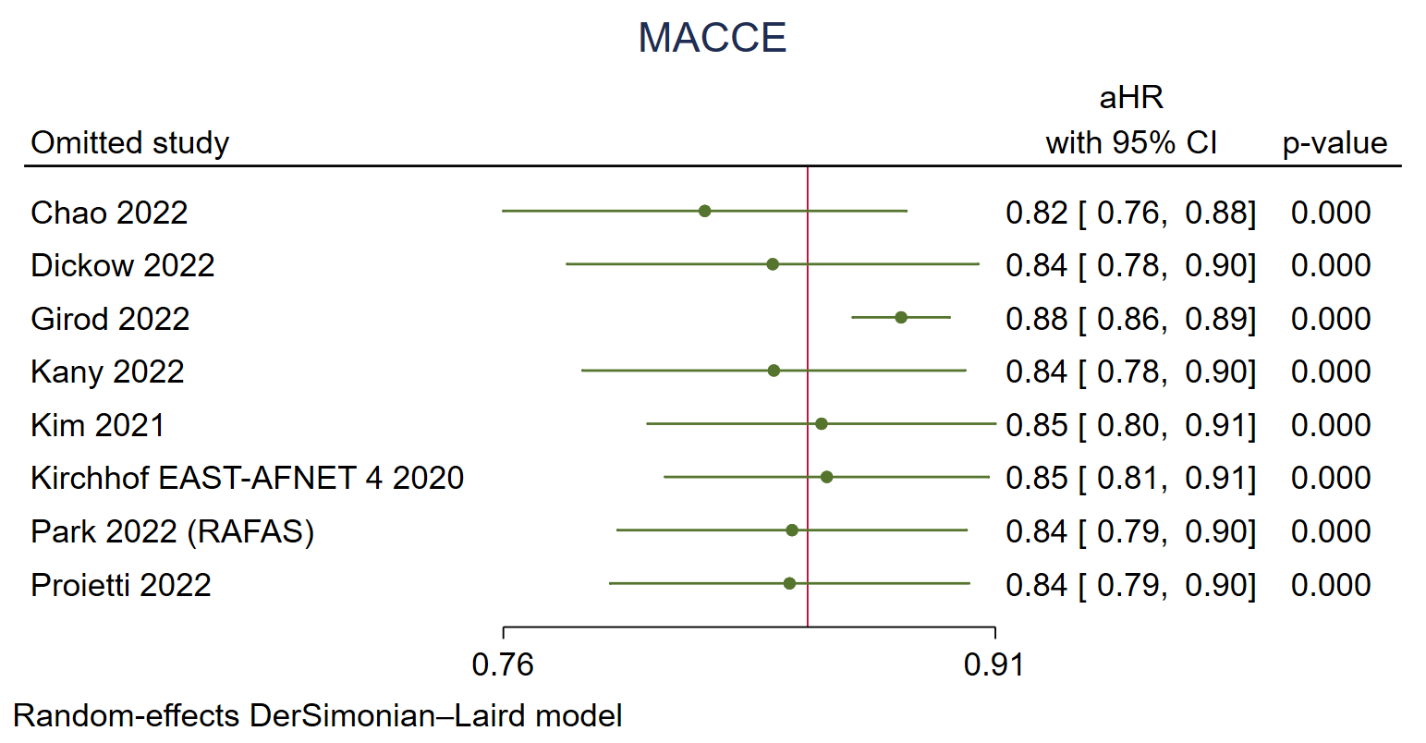


Supplementary Figure 1 Leave-one-out sensitivity analysis for MACCE


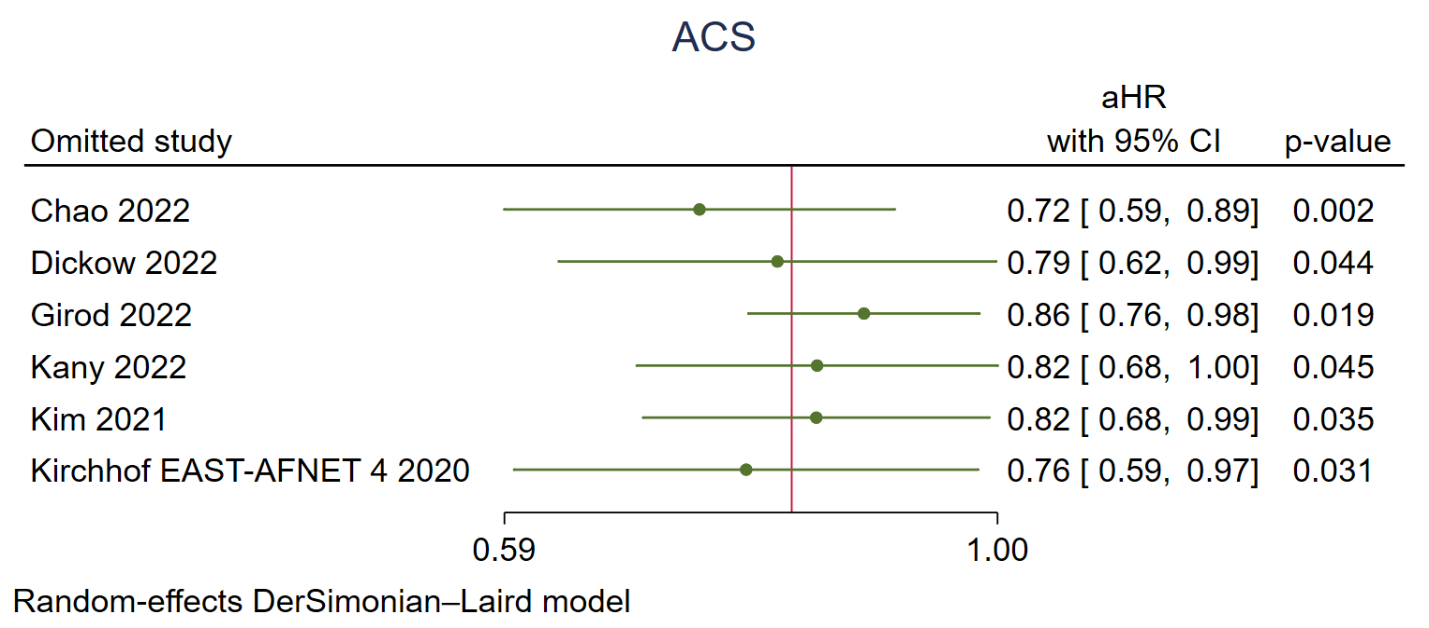


Supplementary Figure 2 Leave-one-out sensitivity analysis for ACS


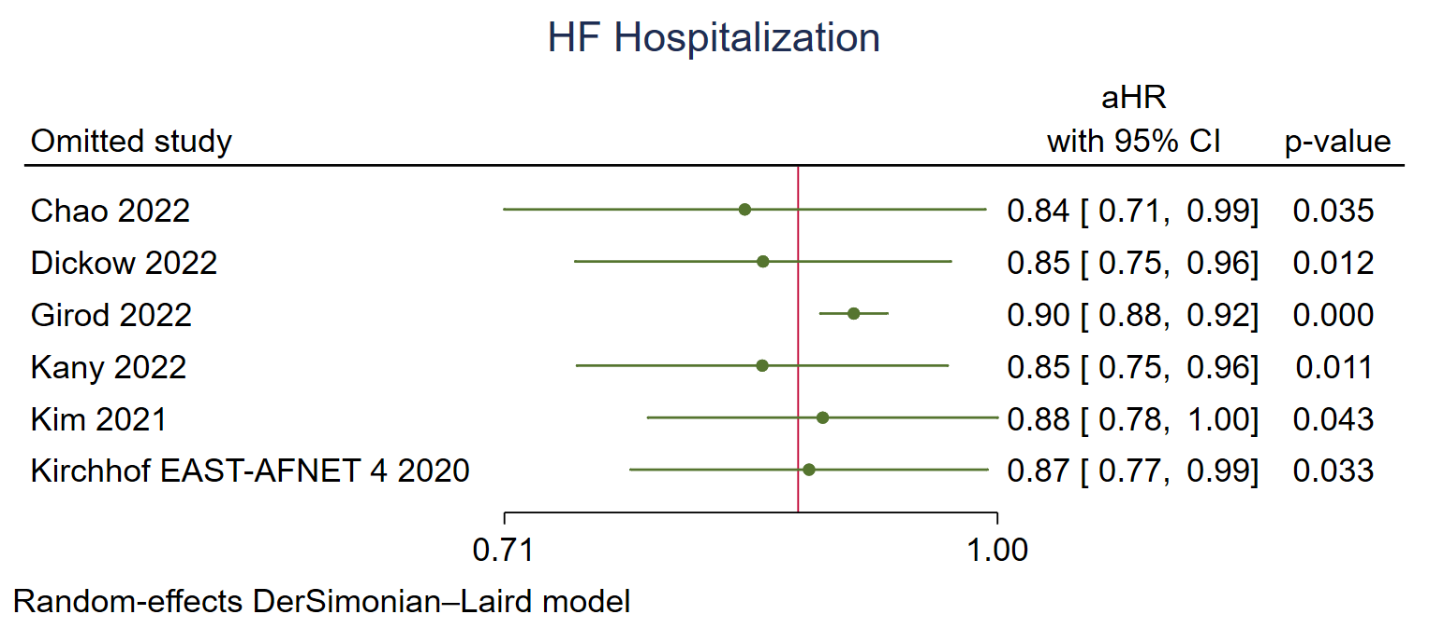


Supplementary Figure 3 Leave-one-out sensitivity analysis for HFH


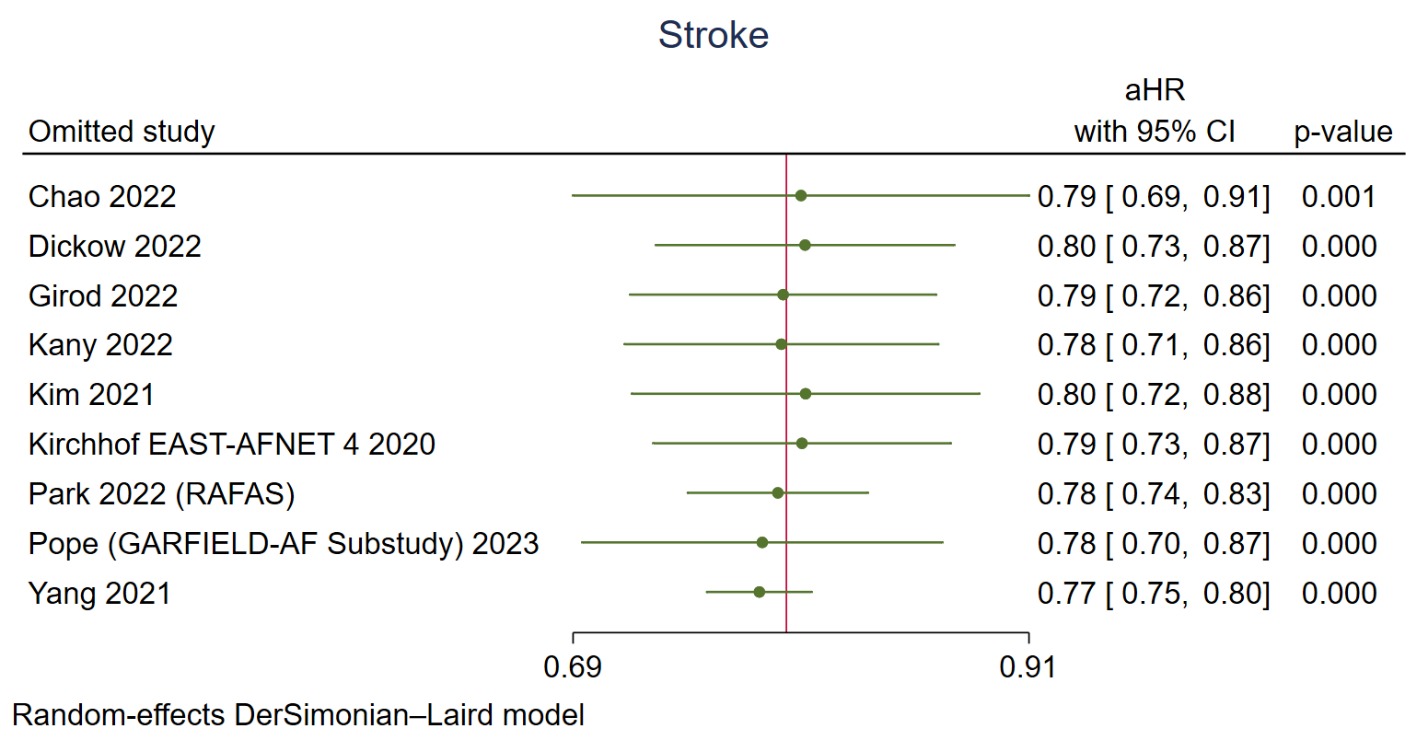


Supplementary Figure 4 Leave-one-out sensitivity analysis for stroke


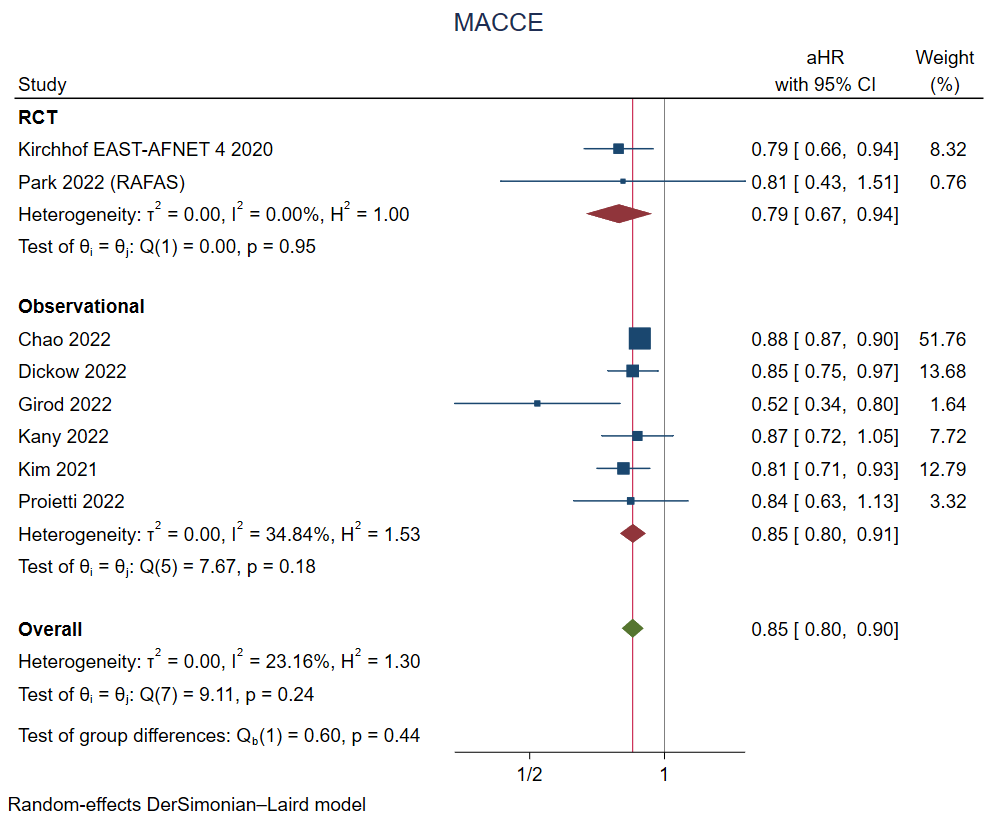


Supplementary Figure 5 Subgroup Analysis based on study design for MACCE


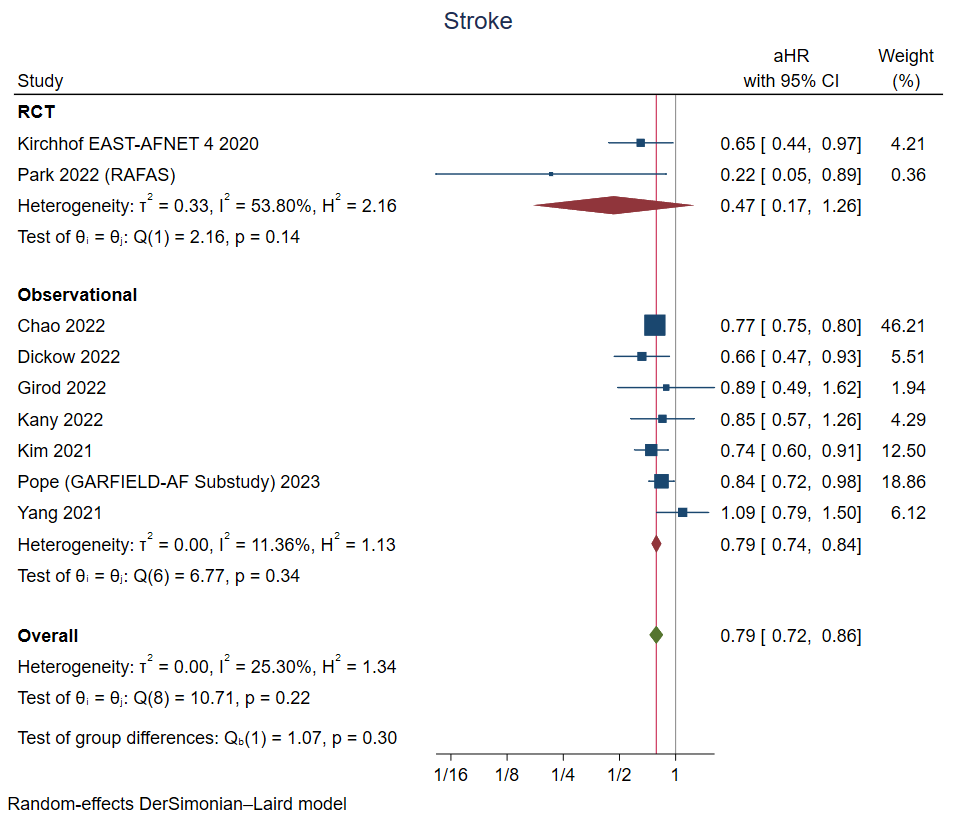


Supplementary Figure 6 Subgroup Analysis based on study design for stroke
